# Supplementary figures and images for: Pattern recognition reveals sex‐dependent neural substrates of sexual perception
Source: Hum Brain Mapp. 2023 Feb 11;44(6):2543–56. doi: 10.1002/hbm.26229 (PMC10028630; doi:10.1002/hbm.26229)

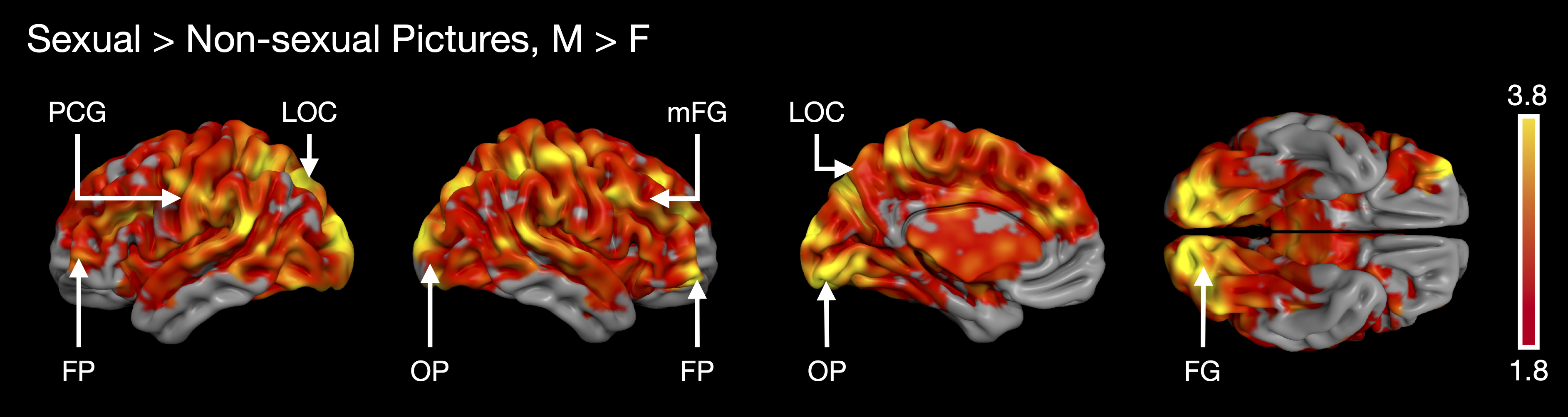

Supplement: Supplementary file 1 — Figure S1. Sex differences (male > female) in response to erotic pictures contrasted with nonerotic pictures depicting humans. The activation maps are thresholded at p < .05, FWE corrected at cluster level. The colourbars represents the t‐value. FG = fusiform gyrus, FP = frontal pole, LOC = lateral occipital cortex, mFG = medial frontal gyrus, OP = occipital pole, PCG = precentral gyrus. [file HBM-44-2543-s005.tiff]

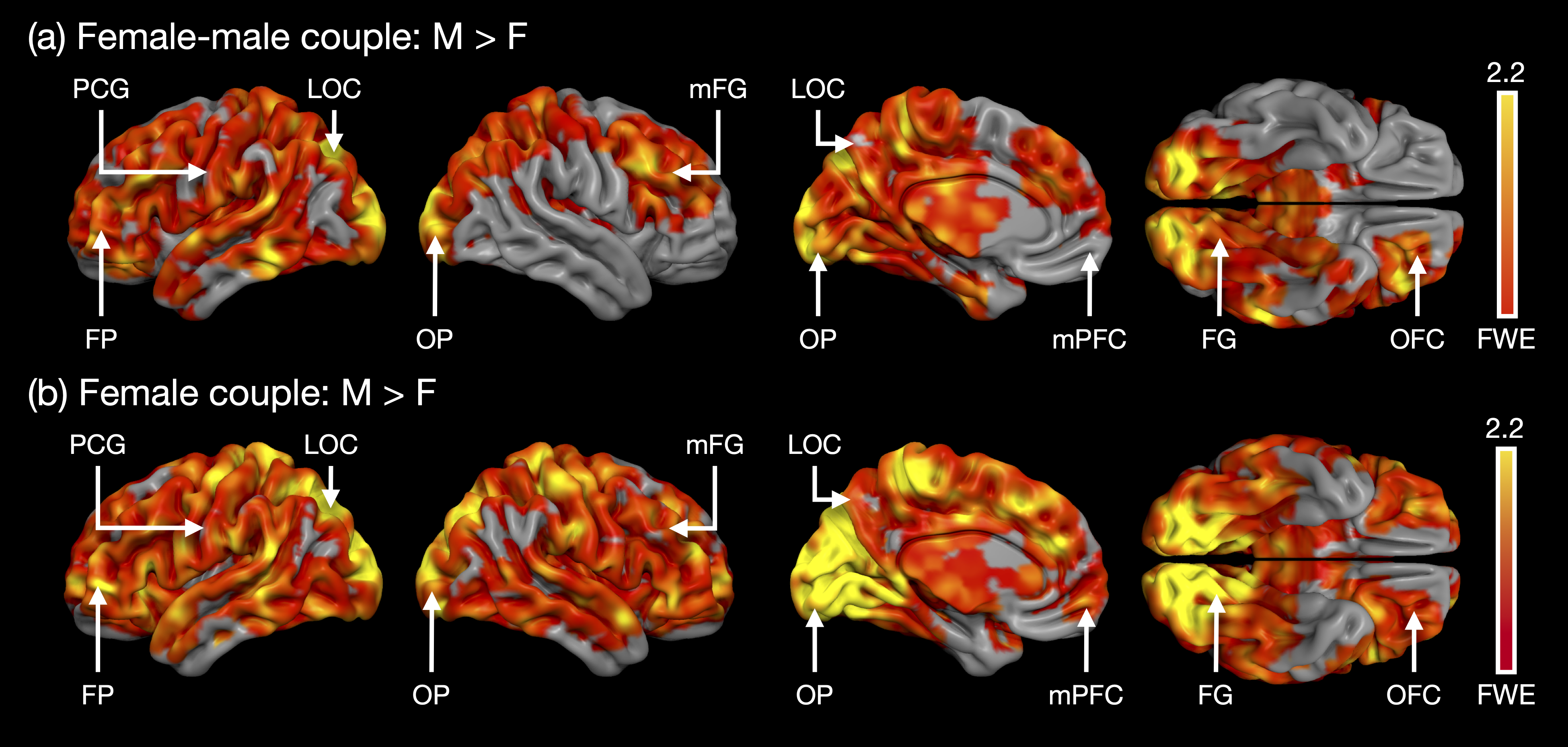

Supplement: Supplementary file 2 — Figure S2. Sex differences (male > female) in response to erotic pictures depicting female–male (a), female–female (b) couples. The activation maps are thresholded at p < .05, FWE corrected at cluster level. The colourbars represents the t‐value. FG = fusiform gyrus, FP = frontal pole, LOC = lateral occipital cortex, mFG = medial frontal gyrus, mPFC = medial prefronal cortex, NAc = nucleus accumbens, OFC = orbitofrontal cortex, OP = occipital pole. [file HBM-44-2543-s001.tiff]

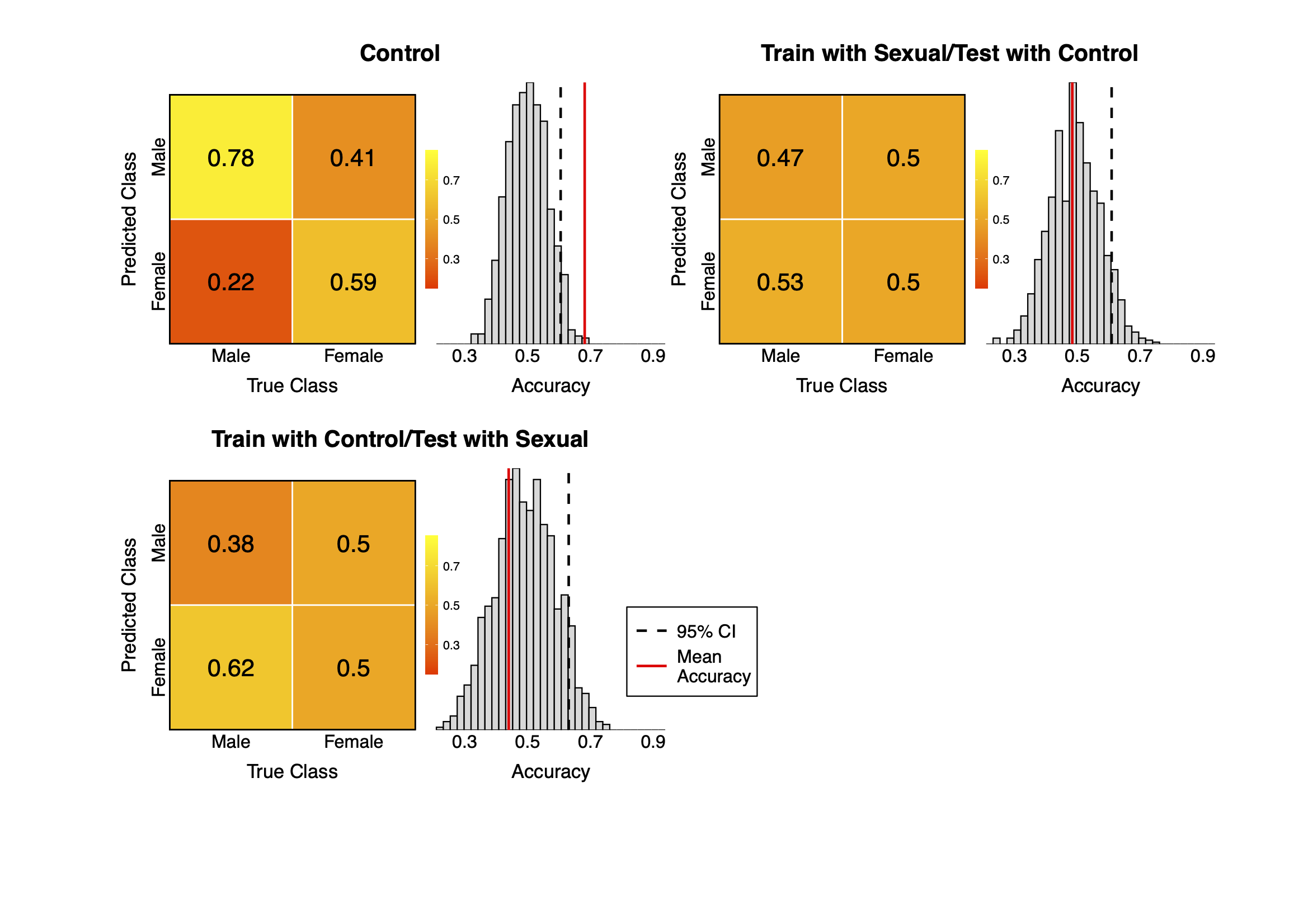

Supplement: Supplementary file 3 — Figure S3. The confusion matrices and permutation results for the sex classification for the control dimension in the movie experiment and the cross‐classification across the control and sexual content dimensions. The numbers in the confusion matrices indicate the proportions of true and false predictions for males and females. The histograms show the null distribution for the classification accuracy. The red vertical lines indicates the mean classification accuracy, and the dashed vertical line the upper confidence interval limit (95% quantile) of the null distribution. [file HBM-44-2543-s006.tiff]

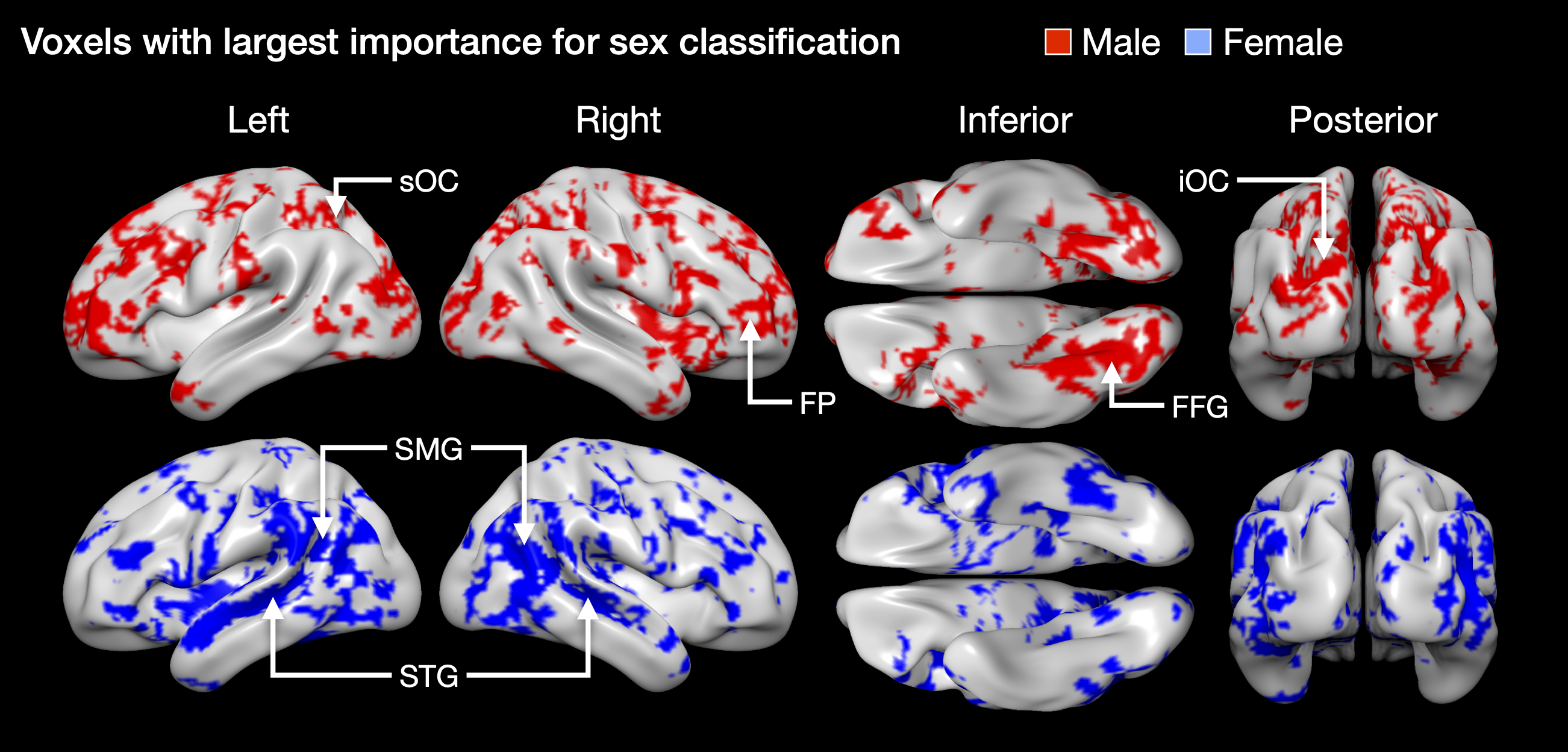

Supplement: Supplementary file 4 — Figure S4. Voxels with the highest importance for the sex‐classification in the movie experiment. The red regions depict voxels that were most indicative of male category and the blue regions depict voxels that were most indicative of female category. For both categories the top 40% of the voxels are shown. FP = Frontal pole, FFG = Fusiform gyrus, SMG = Supramarginal gyrus, STG = Superior temporal gyrus, iOC = inferior occipital cortex, sOC = superior occipital cortex. [file HBM-44-2543-s003.tiff]

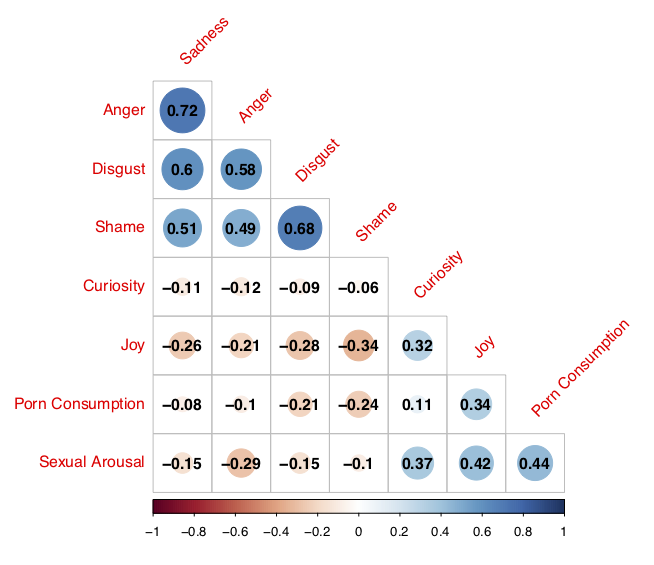

Supplement: Supplementary file 5 — Figure S5. Correlations between self‐ratings of emotions evoked by pornography [file HBM-44-2543-s004.tiff]
