# Supplementary material for: Pattern recognition reveals sex‐dependent neural substrates of sexual perception
Source: Hum Brain Mapp. 2023 Feb 11;44(6):2543–56. doi: 10.1002/hbm.26229 (PMC10028630; doi:10.1002/hbm.26229)
Supplement: Supplementary file 6 — Appendix S1: Supplementary Information [file HBM-44-2543-s002.docx]

**Supplementary Materials for**

**Pattern recognition reveals sex-dependent neural substrates of sexual arousal**

Vesa Putkinen, Sanaz Nazari-Farsani, Tomi Karjalainen, Severi Santavirta, Matthew Hudson, Kerttu Seppälä, Lihua Sun, Henry K. Karlsson, Jussi Hirvonen, Lauri Nummenmaa

**Brain responses to sexual pictures vs. non-sexual pictures of humans**

Males showed stronger responses than women in the same regions as in the main GLM analysis for the pictures experiment (Figure S1, Figure 4b). No region showed stronger activity in females than in males.

**… insert Figure S1 here ----**

**Figure S1.** Sex differences (male > female) in response to erotic pictures contrasted with non-erotic pictures depicting humans. The activation maps are thresholded at *P* < 0.05, FWE corrected at cluster level. The colourbars represents the *t*-value. FG = fusiform gyrus, FP = frontal pole, LOC = lateral occipital cortex, mFG = medial frontal gyrus, OP = occipital pole, PCG = precentral gyrus.

**Brain responses to pictures of female-male and female-female couples**

Separate analyses for the erotic picture categories indicated that males showed stronger activity in responses to both the pictures with male-female and female-female couples (**Supplementary Figure S2**). Female subject did not show stronger activity than the males for neither of the erotic picture categories.

**… insert Figure S2 here ----**

**Figure S2.** Sex differences (male > female) in response to erotic pictures depicting female-male (a), female-female (b) couples. The activation maps are thresholded at *P* < 0.05, FWE corrected at cluster level. The colourbars represents the *t*-value. FG = fusiform gyrus, FP = frontal pole, LOC = lateral occipital cortex, mFG = medial frontal gyrus, mPFC = medial prefronal cortex, NAc = nucleus accumbens, OFC = orbitofrontal cortex, OP = occipital pole.

**Multivariate pattern classification: Control dimension**

**… insert Figure S3 here ----**

**Figure S3.** The confusion matrices and permutation results for the sex classification for the control dimension in the movie experiment and the cross-classification across the control and sexual content dimensions. The numbers in the confusion matrices indicate the proportions of true and false predictionsfor males and females. The histograms show the null distribution for the classification accuracy. The red vertical lines indicates the mean classification accuracy, and the dashed vertical line the upper confidence interval limit (95% quantile) of the null distribution.

**… insert Figure S4 here ----**

**Figure S4.** Voxels with the highest importance for the sex-classification in the movie experiment. The red regions depict voxels that were most indicative of male category and the blue regions depict voxels that were most indicative of female category. For both categories the top 40% of the voxels are shown. FP = Frontal pole, FFG = Fusiform gyrus, SMG = Supramarginal gyrus, STG = Superior temporal gyrus, iOC = inferior occipital cortex, sOC = superior occipital cortex.

**Questionnaire responses of correctly classified and misclassified subjects**

We tested whether misclassified subjects would show more sex-atypical emotional responses to pornogaphy. A repeated measures analysis of variance (ANOVA) of questionnaire data revealed a significant Emotion × Sex × Correctly vs Misclassfied interaction (F(7,483) = 3.767, p < .01) on the ratings for emotion elicited by pornography. According to post-hoc pair-wise comparisons this interaction resulted from the misclassified males showing less anger, disgust, sadness and shame than the correctly classified males (all p < .05). No differences were found between the correctly vs. misclassified females. The corresponding ANOVAs on the actual and desired frequency of different sexual activities (Derogatis, 1979) did not show any differences between the correcly classified and misclassified subjects.

**… insert Figure S5 here ----**

**Figure S5.** Correlations between self-ratings of emotions evoked by pornography
